# Supplementary material for: Use of surrogate endpoints in health technology assessment: a review of selected NICE technology appraisals in oncology
Source: Int J Technol Assess Health Care. 2025 Feb 19;41(1):e11. doi: 10.1017/S0266462325000017 (PMC11894387; doi:10.1017/S0266462325000017)
Supplement: Wheaton and Bujkiewicz supplementary material [file S0266462325000017sup001.docx]

Table 1: Extracted information from TAs

| TA | Surrogate | Final | Level of evidence | Was association between surrogate and final investigated? | Method to estimate association | Measure of association | Was association between treatment effects on surrogate endpoint and final outcome investigated? | Method to estimate association | Measure of association | Were predictions of treatment effect considered? | Method for prediction | Measure of prediction |
| --- | --- | --- | --- | --- | --- | --- | --- | --- | --- | --- | --- | --- |
| TA885 | PFS | OS | Biological plausibility | Yes | Examines relationship between TTP/PFS and PPS in pivotal trial Keynote-826 - exact method is not available in documents | "positive correlation" - exact correlation is not available in documents | No | NA | NA | No | NA | NA |
| TA876 | pCR | EFS | RCT | Yes | Company referenced following papers to support surrogate relationship:  Waser et al (abstract only): MA of RCTs, NRS and and cohorts treated with neo-adjuvant therapy (NEO-AT). KM curves digitized and IPD reconstructed to estimate HRs for EFS by pathologic response status (pCR vs no pCR). Random-effects meta-analysis conducted with 2 RCTs, 3 SATs and 28 cohorts.  BMS data on file: UNAVAILABLE BMS data on file: UNAVAILABLE | Waser et al: HRs ranged from 0.26-0.76 and meta-analysed HR across 12 studies was 0.50 (95% CI: 0.40, 0.62). BMS data on file: UNAVAILABLE BMS data on file: UNAVAILABLE | No | NA | NA | No | NA | NA |
|  | pCR | OS | RCT | Yes | Company referenced following papers to support surrogate relationship: Waser et al (abstract only): MA of RCTs, NRS and cohorts treated with neo-adjuvant therapy (NEO-AT). KM curves digitized and IPD reconstructed to estimate HRs for OS by pathologic response states (pCR vs no pCR). Random-effects meta-analysis conducted with 2 RCTs, 3 SATs and 28 cohorts.  BMS data on file: UNAVAILABLE BMS data on file: UNAVAILABLE | Waser et al: HRs ranged from 0.13-0.78 and meta-analysed HR across 21 studies was 0.49 (95% CI: 0.43, 0.56).  BMS data on file: UNAVAILABLE BMS data on file: UNAVAILABLE | No | NA | NA | No | NA | NA |
|  | EFS | OS | RCT | Yes | Company referenced following papers to support surrogate relationship: Ostoros et al (abstract only): SR and MA of 74 sources (18 RCTs, 26 SATs and 30 observational studies) for neo-adjuvant therapy (NEO-AT) including chemotherapy and/or immunotherapy. Extracted outcomes included HRs comparing EFS and OS between treatment arms, median EFS and median OS. Correlation and regression analyses conducted to evaluate association between mEFS and mOS and effect of treatment on EFS and OS using logHRs.  BMS data on file: UNAVAILABLE BMS data on file: UNAVAILABLE | Ostoros et al: 39 studies reported mEFS and mOS and none included immunotherapy. Pearson's correlation coefficient = 0.819 (95% CI: 0.728, 0.922) BMS data on file: UNAVAILABLE BMS data on file: UNAVAILABLE | Yes | Company referenced following papers to support surrogate relationship: Ostoros et al (abstract only): SR and MA of 74 sources (18 RCTs, 26 SATs and 30 observational studies) for neo-adjuvant therapy (NEO-AT) including chemotherapy and/or immunotherapy. Extracted outcomes included HRs comparing EFS and OS between treatment arms, median EFS and median OS. Correlation and regression analyses conducted to evaluate association between mEFS and mOS and effect of treatment on EFS and OS using logHRs.  BMS data on file: UNAVAILABLE BMS data on file: UNAVAILABLE | Ostoros et al: 8 studies reported HRs from RCTs and none included immunotherapy. Positive linear correlation between EFS and OS logHRs weighted r = 0.864 (95% CI: 0.809, 0.992) and strong association between log treatment effects (random effects meta-regression R-sqaured = 0.777) BMS data on file: UNAVAILABLE BMS data on file: UNAVAILABLE | No | NA | NA |
| TA874 | PFS | OS | Observational study | Yes | Company referenced the following paper to support surrogate relationship: Maurer et al: all patients treated with rituximab containing, anthracycline-based immunochemotherapy as part of initial induction therapy included. Investigated survival between patients achieving PFS24 (alive and progression free at 24 months) and those not achieving PFS24. Standardised mortality ratio (SMR) defined as ratio of observed deaths to expected deaths in general population calculated. | Maurer et al: 3678 patients achieved PFS24, SMR after achieving PFS24 was 1.22 (95% CI: 1.09, 1.37). Observed OS vs expected OS at 3, 5 and 7 years after PFS24 was 93.1% vs 94.4%, 87.6% vs 89.5% and 80% vs 83.7%. | No | NA | NA | No | NA | NA |
|  | EFS | OS | Observational study | Yes | Company referenced the following paper to support surrogate relationship: Jakobsen et al: Patients from Danish lymphoma registry newly diagnosed with diffuse large b-cell lymphoma (DLBCL) included. All patients achieved complete remission or complete remission unconfirmed after first-line rituximab plus cyclophophamide, doxorubicin, vinicristine and prednison (R-CHOP) or R-CHOP like therapy. Survival of patients with DLBCL at different pEFS milestones (6, 24, 36, 48 and 60 months) computed using KM method. General population obtained from Danish life tables. Excess mortality in patients with DLBCL was calculated as standardised mortality ratio (SMR). | Jakobsen et al: 5-year pOS lower for patients with DLBCL than for matched population with SMR: 1.75 (95% CI: 1.60, 1.91) but reduced to 1.27 (95% CI: 1.12, 1.44) for patients achieving pEFS24 and 1.32 (95% CI: 1.11, 1.54) for patients achieving pEFS48. Although this population-based study does not support complete normalisation of survival for patients with DLBCL achieving pEFS24, the estimated loss of residual lifetime was low for patients in continuous remission 2 years after ending treatment. Therefore, pEFS24 is an appealing and relevant milestone for patient counseling and could be a surrogate endpoint in clinical trials. | No | NA | NA | No | NA | NA |
| TA862 | PFS | OS | RCT | Yes | Company referenced the following papers to support surrogate relationship:  Beauchemlin et al: SR including 144 studies of women with metastatic breast cancer (mBC) treated with standard treatments for mBC or BSC looking at PFS/TTP and OS. Relationship between PFS/TTP and OS evaluated using Pearson's or Spearman's correlation.  Lui et al (abstract only): Analysis of 24 RCTs in patients treated with second- or third-line chemotherapy for mBC. Spearman's correlation calculated between PFS and OS. | Beauchemlin et al: Unweighted Spearman correlation coefficient for PFS/TTP and OS was 0.428 (p<0.01), correlation was higher for studies using PFS as endpoint: 0.523 (p<0.01).  Lui et al (abstract only): Spearman's correlation between PFS/TTP and OS was 0.7824 (95% CI: 0.6034, 0.8702). | Yes | Company referenced the following papers to support surrogate relationship:  Beauchemlin et al: SR including 144 studies of women with metastatic breast cancer (mBC) treated with stadard treatments for mBC or BSC looking at difference in medians between treatment arms. Pearson's or Spearman's correlation used to evaluate association between difference in medians for PFS/TTP and difference in medians for OS. Adunlin et al: SR and MA including 72 trials in mBC for patients receiving anthracyclines, taxanes or targeted therapies. HRs for PFS and OS obtained from comparative arms. Spearman's correlation and weighted multivariate regression used to test strength of association between HR PFS and HR OS.  Lui et al (abstract only): Analysis of 24 RCTs in patients treated with second- or third-line chemotherapy for mBC. Weighted linear regression conducted between HR PFS/TTP and HR OS. | Beauchemlin et al: Unweighted Spearman correlation coefficient between differences in PFS/TTP and differences in OS estimated at 0.427 (p<0.01).  Adunlin et al: Univariate Spearman correlation coefficient between HR PFS and HR OS estimated at 0.46 (p<0.001). Coefficient on HR PFS in weighted multivariate regression was 0.18 (p=0.04) indicating that it is a significant predictor of HR OS. However, R-squared only 0.31 indicating only some of variability was accounted for. When trials included were limited to 2nd line or higher, the strength of association improved (0.4; p<0.001) and model R-squared increased to 0.55.  Lui et al (abstract only): Association between HRs of PFS/TTP and OS of 22 studies showed moderate correlation (r=0.5725 95% CI:I 0.1735, 0.8277), slope of regression model was 0.5366 (95% CI: 0.3479, 0.7253). PFS/OS correlation for HER2+ mBC patients was stronger (spearman's corr = 0.9515, 95% CI: 0.7009, 1 and regression coefficient = 0.8728, 95% CI: 0.0795, 1.6661). | Yes | Company referenced the following papers to support surrogate relationship:  Beauchemlin et al: SR including 144 studies of women with mBC treated with standard treatments for mBC or BSC looking at difference in medians between treatment arms. Linear regression conducted to predict effects of new anti-cancer drug on OS on basis of effects on PFS/TTP. For this analysis only studies that presented statistically significant difference in PFS/TTP and OS between treatment arms were used to build prediction model. Coefficient of determination (R-squared) calculated to measure proportion of variance in difference in OS explained by variance in difference in PFS/TTP | Beauchemlin et al: 14 studies presented statistically significant difference in PFS/TTP and OS between treatment arms. Regression equation was: diff in OS = -0.088 (95% CI: -1.347, 1.172) + 1.753 (95% CI: 1.307, 2.198) * diff PFS/TTP. R-squared = 0.86. Results of regression analysis predict that difference in median PFS/TTP of 5, 10, 15 and 20 months would tranlate into a difference in median OS of 8.7, 17.4, 26.2 and 35.0 months respectively. |
| TA851 | pCR | EFS | Biological plausibility | No | NA | NA | No | NA | NA | No | NA | NA |
|  | pCR | OS | Biological plausibility | No | NA | NA | No | NA | NA | No | NA | NA |
|  | EFS | OS | Biological plausibility | No | NA | NA | No | NA | NA | No | NA | NA |
| TA837 | RFS | DMFS | RCT | No | NA | NA | Yes | Company referenced the following papers to support surrogate relationship: Eggermont et al: adjuvant ipilumumab versus placebo after complete resection of stage 3 melanoma: long term follow-up results for the phase 3 RCT. However, this just reports long term HRs for RFS, DMFS and OS and the authors of TA argue that the similarity of the HRs across these outcomes suggests support for surrogate relationship. | Eggermont et al: RFS HR: 0.75 (95% CI: 0.63, 0.88), DMFS HR: 0.76 (95% CI: 0.64, 0.90) and HR OS: 0.73 (95% CI: 0.60, 0.89). | No | NA | NA |
|  | RFS | OS | RCT | Yes | Company referenced the following papers to support surrogate relationship: Coart et al: analysed single RCT with IPD to estimate Spearmans correlation between RFS and OS. Suciu et al: SR and MA: IPD collected from 11 RCTs comparing interferon to observation and 2 studies comparing interferon and vaccination in stage 2/3 melanoma patients. Spearmans correlation used to calculate correlation between RFS and OS. | Coart et al: Spearmans correlation from single trial estimated as 0.84 (95% CI: 0.82, 0.87).  Suciu et al: Spearmans correlation from 12 trials estimated to be 0.77. | Yes | Company referenced the following papers to support surrogate relationship:  Coart et al: analysed single RCT using geographic location as unit of analysis. Measured unit-level association between HR RFS and HR OS using weighted linear regression coefficient of determination - R-squared.  Koruth et al (abstract only): SR identified 18 studies investigating dabrafenib + trametinib (D+T), ipilumumab, vemurafenib, chemo and interferons in adjuvant high risk radically resected cutaneous melanoma. Weighted linear regression of logHR RFS and logHR OS and correlation of logHR RFS and logHR OS used to estimate association between treatment effects. Suciu et al: SR and MA: IPD collected from 11 RCTs comparing interferon to observation and 2 studies comparing interferon and vaccination in stage 2/3 melanoma patients. Linear regression on HR RFS and HR OS to estimate trial-level association. | Coart et al: Association from weighted linear regression estimated to be 0.59 (95% CI: 0.08, 1.00).  Koruth et al (abstract only): weighted linear regression was logHR OS = 0.03 + 0.898 logHR RFS and correlation coefficient was 0.74.  Suciu et al: Linear regression equation estimated to be HR OS = exp(0.0106 + 0.9874 * ln(HR RFS)). Indicates that risk reduction due to interferon treatment was approximately the same for RFS and OS. | Yes | Company referenced the following papers to support surrogate relationship: Suciu et al: SR and MA: IPD collected from 11 RCTs comparing interferon to observation and 2 studies comparing interferon and vaccination in stage 2/3 melanoma patients. Surrogate threshold effect was calculated and defined as the minimum value of treatment effect on the surrogate endpoint, for which the predicted effect on the true endpoint would be different from zero. | Suciu et al: Surrogate threshold effect corresponded to HR for RFS of 0.77. Thus in a future adjuvant trial, in order to predict a positive treatment effect on OS, a hazard ratio fro RFS of 0.77 or less would need to be achieved. |
|  | DMFS | OS | Biological plausibility | No | NA | NA | No | NA | NA | No | NA | NA |
| TA830 | DFS | OS | Biological plausibility | No | NA | NA | No | NA | NA | No | NA | NA |
| TA833 | VGPR/CR | PFS | No discussion of validation | No | NA | NA | No | NA | NA | No | NA | NA |
|  | VGPR/CR | OS | No discussion of validation | No | NA | NA | No | NA | NA | No | NA | NA |
| TA823 | DFS | OS | RCT | No | NA | NA | Mauguen et al: squared correlations between | Company referenced the following paper to support surrogate relationship: Mauguen et al: SR and MA of 24 trials (for DFS) evaluating chemotherapy and radiotherapy in operable and locally advanced lung cancer. Weighted linear regression used to calculate trial-level association between HR on DFS and HR on OS. Squared correlation calculated between HR on DFS and HR on OS | Mauguen et al: Squared correlation between HR on DFS and HR on OS were 0.92 (95% CI: 0.88, 0.95) for chemo vs no chemo and 0.99 (95% CI: 0.98, 1) for radio + chemo vs radio alone. Weighted linear regression logHR OS = 0.08 + 0.98 * logHR PFS | Yes | Company referenced the following paper to support surrogate relationship:  Mauguen et al: SR and MA of 24 trials (for DFS) evaluating chemotherapy and radiotherapy in operable and locally advanced lung cancer. Surrogate threshold effect (STE) calculated as minimum effect on surrogate that would be necessary to predict a non-zero effect on survival. | Mauguen et al: STE estimated as 0.88 for chemotherapy compared to no chemotherapy and 0.95 for radiotherapy plus chemotherapy compared to radiotherapy alone. |
| TA817 | DFS | OS | RCT | No | NA | NA | No | NA | NA | No | NA | NA |
| TA813 | MMR at 24 weeks | OS | Observational study | Yes | Company referenced the following paper to support surrogate relationship: Ciani et al: Pooled weighted mean OS estimated by MMR response or not at time periods 12, 24, 36, 48, 60, 72 and 84 months after starting of imatinib therapy. | Ciani et al: Pooled weighted mean OS for MMR at 24 months is 100 and 96.7 for no MMR. | No | NA | NA | No | NA | NA |
|  | CyR | PFS | No discussion of validation | No | NA | NA | No | NA | NA | No | NA | NA |
|  | CyR | OS | No discussion of validation | No | NA | NA | No | NA | NA | No | NA | NA |
|  | CCyR | PFS | No discussion of validation | No | NA | NA | No | NA | NA | No | NA | NA |
|  |  | OS | Observational study | Yes | Company referenced the following paper to support surrogate relationship: Ciani et al: Pooled weighted mean OS estimated by CCyR response or not at time periods 12, 24, 36, 48, 60, 72 and 84 months after starting of imatinib therapy. | Ciani et al: Pooled weighted mean OS for CCyR at 24 months is 98.3 for CCyR and 94 for no CCyR. | No | NA | NA | No | NA | NA |
|  | TTD | PFS | Biological plausibility | No | NA | NA | No | NA | NA | No | NA | NA |
|  |  | OS | Biological plausibility | No | NA | NA | No | NA | NA | No | NA | NA |
| TA810 | IDFS | OS | Biological plausibility | No | NA | NA | No | NA | NA | No | NA | NA |
|  | DRFS | OS | Biological plausibility | No | NA | NA | No | NA | NA | No | NA | NA |
| TA796 | ToT/TTD | PFS | No discussion of validation | No | NA | NA | No | NA | NA | No | NA | NA |
| TA795 | ToT/TTD | PFS | Observational study | No | NA | NA | No | NA | NA | No | NA | NA |
| TA784 | PFS | OS | Biological plausibility | No | NA | NA | No | NA | NA | No | NA | NA |
| TA772 | PFS | OS | Biological plausibility | No | NA | NA | No | NA | NA | No | NA | NA |
| TA763 | MRD | PFS | RCT | Yes | Company referenced the following paper to support surrogate relationship:  Rawstron et al: Analysis of single RCT (MRC Myeloma IX) involving 397 patients randomly assigned to cyclophosphamide, thalidomide and dexamethason (CTD) or cyclophosphamide, vinicristine, doxorubicin and dexamethasone (CVAD) and then high dose melphalan and autologous stem cell transplant (ASCT). Univariate and multivariate Cox PH model applied to PFS data with log MRD as a predictor.  de Tute et al (abstract only): Analysis of single RCT (Myeloma XI) for transplant ineligible MM patients. Multivariate analysis (does not specify model) applied to PFS data with log MRD as a predictor.  Munshi et al: Review and MA of 14 studies in newly diagnosed multiple myeloma (NDMM) patients receiving any therapy other than allogeneic stem cell transplant (ASCT). Stratified analysis by MRD status to investigate impact of MRD status on HR PFS. | Rawstron et al: states that level of MRD is an independent predictor of PFS (p-value<0.0001) de Tute et al (abstract only): states that log MRD is an independent predictor of PFS (p=0.004).  Munshi et al: Compared with MRD+, MRD- was associated with better PFS (HR: 0.41 95% CI: 0.36, 0.48). | Yes | Company referenced following papers to support surrogate relationship: Avet-Loiseau et al: SR and MA of six RCTs of patients with newly diagnosed multiple myeloma (NDMM). Weighted linear regression used to estimate association between odds ratios (OR) calculated based on MRD negativity rate and HRs on PFS. R-squared was also calculated for this weighted linear regression. | Avet-Loiseau et al: weighted linear regression estimated as logHR PFS = -0.4 * logOR MRD - 0.09. R-squared for weighted linear regression estimated to be 0.97. | No | NA | NA |
|  | MRD | OS | RCT | Yes | Company referenced the following papers to support surrogate relationship:  Rawstron et al: Analysis of single RCT (MRC Myeloma IX) involving 397 patients randomly assigned to CTD or CVAD followed by high-dose melphalan and ASCT. Univariate and multivariate cox PH model applied to OS data with log MRD as predictor.  Company referenced following papers to support surrogate relationship: Munshi et al: Review and MA of 12 studies in newly diagnosed multiple myeloma (NDMM) patients receiving any therapy other than allogeneic stem cell transplant (ASCT). Stratified analysis by MRD status to investigate impact of MRD status on HR OS. | Rawstron et al: States that level of MRD is an independent predictor of OS (p value < 0.001).  Munshi et al: compared with MRD+, MRD- was associated with better OS (HR: 0.57, 95% CI: 0.46, 0.71). | No | NA | NA | No | NA | NA |
|  | sCR | PFS | Observational study | Yes | Company referenced the following paper to support surrogate relationship:  Kapoor et al: observational study of 445 patients who underwent autologous stem cell transplant (ASCT) within 12 months diagnosis of multiple myeloma. Simple comparison of median TTP from those achieving sCR compared to those achieving CR or nCR. | Kappor: States that median TTP of patients achieving sCR was significantly longer (50 months) than TTP of patients achieving CR or nCR (20 months and 19 months respectively). | No | NA | NA | No | NA | NA |
|  | sCR | OS | Observational study | Yes | Company referenced the following paper to support surrogate relationship: Kapoor et al: observational study of 445 patients who underwent autologous stem cell transplant (ASCT) within 12 months diagnosis of multiple myeloma. Univariate and multivariate cox model on OS for sCR vs other responses (complete response (CR), near complete response (nCR), very good partial response (VGPR), partial response (PR), stable disease (SD) and progressive disease (PD) conducted. | Kapoor et al: post-ASCT response of sCR was an independent prognostic factor for survival: HR 0.44 (95% CI: 0.25, 0.80). | No | NA | NA | No | NA | NA |
| TA766 | RFS | OS | RCT | Yes | Company referenced the following papers to support surrogate relationship: Coart et al: analysed single RCT with IPD to estimate Spearmans correlation between RFS and OS. Suciu et al: SR and MA: IPD collected from 11 RCTs comparing interferon to observation and 2 studies comparing interferon and vaccination in stage 2/3 melanoma patients. Spearmans correlation used to calculate correlation between RFS and OS. | Coart et al: Spearmans correlation from single trial estimated as 0.84 (95% CI: 0.82, 0.87).  Suciu et al: Spearmans correlation from 12 trials estimated to be 0.77. | Yes | Company referenced the following papers to support surrogate relationship:  Coart et al: analysed single RCT using geographic location as unit of analysis. Measured unit-level association between HR RFS and HR OS using weighted linear regression coefficient of determination - R-squared.  Koruth et al (abstract only): SR identified 18 studies investigating dabrafenib + trametinib (D+T), ipilumumab, vemurafenib, chemo and interferons in adjuvant high risk radically resected cutaneous melanoma. Weighted linear regression of logHR RFS and logHR OS and correlation of logHR RFS and logHR OS used to estimate association between treatment effects. Suciu et al: SR and MA: IPD collected from 11 RCTs comparing interferon to observation and 2 studies comparing interferon and vaccination in stage 2/3 melanoma patients. Linear regression on HR RFS and HR OS to estimate trial-level association. | Coart et al: Association from weighted linear regression estimated to be 0.59 (95% CI: 0.08, 1.00).  Koruth et al (abstract only): weighted linear regression was logHR OS = 0.03 + 0.898 logHR RFS and correlation coefficient was 0.74.  Suciu et al: Linear regression equation estimated to be HR OS = exp(0.0106 + 0.9874 * ln(HR RFS)). Indicates that risk reduction due to interferon treatment was approximately the same for RFS and OS. | Yes | Company referenced the following papers to support surrogate relationship: Suciu et al: SR and MA: IPD collected from 11 RCTs comparing interferon to observation and 2 studies comparing interferon and vaccination in stage 2/3 melanoma patients. Surrogate threshold effect was calculated and defined as the minimum value of treatment effect on the surrogate endpoint, for which the predicted effect on the true endpoint would be different from zero. | Suciu et al: Surrogate threshold effect corresponded to HR for RFS of 0.77. Thus in a future adjuvant trial, in order to predict a positive treatment effect on OS, a hazard ratio fro RFS of 0.77 or less would need to be achieved. |
